# The Evolving Global Burden of Young‐Onset Parkinson's Disease (1990–2021): Regional, Gender, and Age Disparities in the Context of Rising Incidence and Declining Mortality

**DOI:** 10.1002/brb3.70659

**Published:** 2025-07-07

**Authors:** Yanjie Jiang, Hanyu Wang, Xingyi He, Rui Fu, Zhihui Jin, Qinwei Fu, Xinlang Yu, Wenshan Li, Xiaoyu Zhu, Shipeng Zhang, Yan Lu

**Affiliations:** ^1^ Nanjing Hospital of Chinese Medicine Affiliated to Nanjing University of Chinese Medicine Nanjing China; ^2^ Hospital of Chengdu University of Traditional Chinese Medicine Chengdu University of Traditional Chinese Medicine Chengdu China; ^3^ The First Affiliated Hospital of Guangzhou University of Chinese Medicine Guangzhou China; ^4^ Department of Health Research Methods, Evidence, and Impact (HEI) McMaster University Hamilton Canada

**Keywords:** age‐standardized rate, estimated annual percentage change, global burden of disease, socio‐demographic index, young‐onset Parkinson's disease

## Abstract

**Background:**

Young‐onset Parkinson's disease (YOPD) has emerged as a significant global public health challenge characterized by its early onset among individuals in their prime working years, complex disruptions to social roles, and a substantial socioeconomic burden encompassing diminished productivity, escalating healthcare costs, and markedly reduced quality of life.

**Methods:**

Using Global Burden of Disease (GBD) 2021 data, this study analyzed YOPD burden (ages 20–49) from 1990 to 2021, assessing age‐standardized incidence rate (ASIR), mortality rate (ASMR), disability‐adjusted life years rate (ASDR), and trends via Bayesian Age‐Period Cohort (BAPC) modeling.

**Results:**

Over the past three decades, the ASIR (estimated annual percentage change [EAPC] = 1.40%) and ASDR (EAPC = 0.14%) have significantly increased globally in YOPD, whereas the ASMR (EAPC = −0.42%) has declined. The sociodemographic index (SDI) exhibits an inverted U‐shaped correlation with the burden of disease, with the middle SDI region representing the core burden zone globally. The rising trend in burden of disease is most pronounced in the middle SDI, and the high‐middle SDI has the most significant upward trend in disease burden. Notably, East, South, and Southeast Asia accounted for more than 60% of global cases and deaths, led by China and India. Additionally, the burden of YOPD increases with age, with the number of new YOPD cases, deaths, and disability‐adjusted life years (DALYs) being 48%, 92%, and 73% higher in men than in women, respectively. By 2050, the ASIR is projected to rise by 12.39%, whereas the ASMR and ASDR may drop by 23.75% and by 22.05%, reflecting a “rising incidence paired with declining mortality and projected reductions in disability burden” trend.

**Conclusion:**

YOPD disproportionately impacts working‐age populations, with geographic, gender, and age disparities. This analysis establishes YOPD as a priority condition for global neurological health initiatives, particularly in middle SDI regions and Asia, requiring differentiated regional approaches to mitigate its growing impact on working‐age populations.

## Graphical abstract

This study highlights the rising global burden of young‐onset Parkinson's disease (YOPD), focusing on regional, gender, and age disparities. The increasing incidence contrasts with declining mortality, indicating the need for targeted interventions, particularly in Asia and Middle SDI regions, to alleviate the disease burden among essential global workforce cohorts.

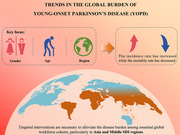

**Abbreviations**
ASDR: age‐standardized disability‐adjusted life year rate
ASIR: age‐standardized incidence rate
ASMR: age‐standardized mortality rate
ASR: age‐standardized rate
CI: confidence interval
DALYs: disability‐adjusted life‐years
EAPC: estimated annual percentage change
EOPD: early ‐onset Parkinson's disease
GBD: Global Burden of Disease study
LOPD: late‐onset Parkinson's disease
PD: Parkinson's disease
SDI: sociodemographic index
UI: uncertainty interval
YOPD: young‐onset Parkinson's disease

## 1 Introduction

Parkinson's disease (PD) is one of the fastest growing neurological disorders in the world and is pathologically characterized by loss of nigrostriatal dopaminergic neurons and abnormal aggregation of α‐synuclein (Morris et al. 2024; Ben‐Shlomo et al. 2024). Although the traditional view is that PD mainly involves older age groups, about 5%–10% of the cases have onset between the ages of 21 and 50 years and are referred to as young‐onset Parkinson's disease (YOPD) (Schrag and Schott 2006). YOPD is gradually becoming a research hotspot in the field of neuroscience due to its distinctive clinical features, socioeconomic impact, and etiological heterogeneity (Santos‐García et al. 2023; Kapelle et al. 2024). Compared with late‐onset Parkinson's disease (LOPD), patients with YOPD develop significant motor dysfunction earlier despite slower progression of motor symptoms (Schrag and Schott 2006; Wickremaratchi et al. 2011). Notably, YOPD patients are mostly in their prime career years or at the peak of their family responsibilities, and their disease‐induced loss of labor productivity far exceeds that of the LOPD group. The average annual indirect economic burden of YOPD patients in the United States has been estimated to be significantly higher than that of LOPD, and the total economic burden of PD is projected to exceed $79 billion by 2037 (Yang et al. 2020). Early cross‐sectional studies have shown that age at onset of YOPD is negatively associated with emotional well‐being and quality of life, with earlier onset of the disease correlating with worse physical and mental states, and the resulting family and social burdens being more pronounced (Knipe et al. 2011; Schrag et al. 2003). Despite the relatively slow progression and long duration of YOPD, the mortality rate of patients with YOPD can be more than five times that of the general population, and the reduction in life expectancy is more pronounced than that of patients with LOPD, with the risk of death being higher in men than in women (Mehanna and Jankovic 2019; Hustad et al. 2021). Because of their age of onset, patients with YOPD face multiple challenges over time, with decades of accumulated disability, as well as career interruptions, changes in family roles, and long‐term healthcare expenditures (Mehanna and Jankovic 2019; Liu et al. 2022).

Previous studies have primarily focused on the overall PD population, whereas epidemiological studies of YOPD have long suffered from data fragmentation. Regional studies have indicated that YOPD is more prevalent among Asian populations than in Europe and the United States, with 15% of PD patients in Japan having disease onset before the age of 50, compared to 5%–7% in Europe and the United States (Golbe 1991; Van Den Eeden et al. 2003). An earlier cross‐sectional study based on people with disabilities in the United States reported a YOPD prevalence of 414.9/100,000, predominantly among white males (48.9%) (Willis et al. 2013). This geographic variation may be attributed to the evolution of diagnostic criteria (e.g., the spread of genetic testing technology has improved the identification of early onset patients) (Kukkle et al. 2022) and the distribution of environmental risk factors (e.g., regional imbalances in pesticide exposure and heavy metal contamination) (Aravindan and Newell 2024). The Global Burden of Disease 2021 (GBD 2021) study reported the global age‐standardized incidence and prevalence of PD as 15.63/100,000 and 138.63/100,000, respectively, but precise data for YOPD were not independently reported (Luo et al. 2024). A recent meta‐analysis of 50 studies addressed this gap, reporting the global age‐standardized incidence and prevalence of YOPD as 1.3/100,000 and 10.2/100,000, respectively (Nabizadeh et al. 2024). The results are constrained by several factors contributing to the heterogeneity of the primary studies, including variations in diagnostic criteria across regions, which may lead to inconsistencies in identifying and classifying YOPD cases. The absence of standardized diagnostic protocols in certain areas further exacerbates these inconsistencies, whereas the quality and comprehensiveness of regional registry systems vary markedly. Some regions may lack robust data collection mechanisms or experience underreporting, potentially biasing prevalence and incidence estimates. Additionally, geographical and sociodemographic disparities in healthcare access and reporting introduce further biases, particularly in less‐developed regions where diagnostic facilities and disease awareness may be limited. Collectively, these factors underscore the limitations of the data and the challenges of generalizing findings across diverse populations. Thus, although the results offer valuable insights, it is crucial to account for these limitations when interpreting the findings.

Although the number of YOPD studies has increased in recent years, most existing studies are limited to cross‐sectional designs or single‐country data, with a lack of cross‐cultural comparisons and long‐term cohort studies. The uneven geographic coverage of epidemiologic data may undermine the reliability of extrapolating global trends. On the basis of the latest data of GBD 2021, this study systematically assesses the evolution of the disease burden of YOPD patients over the last 30 years, considering factors such as sociodemographic index (SDI, a measure used to assess the development level of a country or region), region, country, age group, and gender. It aims to construct a comprehensive system of epidemiological parameters for YOPD, deepen our understanding of its geographic and demographic distribution, and provide scientific evidence to shift PD prevention and control from a “population‐wide intervention” approach to “age‐stratified precise management.”

## 2 Methods

### 2.1 Data Collection

This study presents a retrospective analysis of data from the GBD 2021 Study, retrieved from the Global Health Data Exchange (GHDx) query platform (https://vizhub.healthdata.org/gbd‐results/). The study employed a standardized assessment methodology to systematically analyze population health indicators across 204 countries and territories from 1990 to 2021, incorporating data on census, fertility, disease prevalence, mortality, and other health‐related factors. GBD 2021 utilizes multidimensional data modeling through spatiotemporal Gaussian process regression, applying smoothing to the age, time, and geographic dimensions for regions with incomplete data. The study employed a meta‐regression technique (MR‐BRT) to integrate Bayesian priors, regularization, and data pruning methods, effectively correcting for systematic bias introduced by differences in case definitions and study methodologies. This analysis utilized only de‐identified population health data and did not involve personally identifiable information or animal testing. All analyses adhere to the norms of accuracy and transparency in health assessment reporting, focusing on core indicators such as disease incidence, mortality, and disability‐adjusted life years (DALYs).

### 2.2 Definition of YOPD and Study Population

Early ‐ onset Parkinson's disease (EOPD) is typically defined as a condition characterized by the onset of symptoms before the age of 50 years (Kolicheski et al. 2022). YOPD, an important subcategory of EOPD, is commonly defined in existing literature as having an onset between 21 and 50 years of age (Santos‐García et al. 2023; Liu et al. 2022; Parkinson's Disease and Movement Disorders Group of Neurology Branch of Chinese Medical Association and Parkinson's Disease and Movement Disorders Group of Neurologist Branch of Chinese Physician Association 2021). However, there is no current international academic consensus regarding the terminology and age thresholds for YOPD versus EOPD. Moreover, the age threshold for YOPD has varied considerably in the existing literature, with definitions ranging from ≤40 to ≤50 years (Mehanna and Jankovic 2019; Mehanna et al. 2022). To systematically analyze the clinical characteristics of the early onset population, the term “YOPD” was adopted in this study to emphasize the unique clinical management, genetic background, and social needs of this population, as well as their significant family roles and social responsibilities, which impose specific demands on disease management. Because the GBD database provides data in 5‐year age intervals (e.g., 20–24, 25–29, …, 45–49, 50–54), we defined YOPD as onset between 20 and 49 years of age. By initiating the lower bound at 20 years rather than 21 and excluding the 50–54 interval, our selected age range (20–49 years) aligns precisely with the GBD categorization, thereby encompassing all cases that would be classified as “<50 years” without including those aged ≥50 years. Subjects were individuals with PD who met the criteria outlined in the International Classification of Diseases (ICD), using the 9th/10th revision codes (332‐332.0 and F02.3, G20‐G20.9, respectively). According to the International PD and Movement Disorders Society (MDS), the core feature of PD is “motor parkinsonism,” characterized by bradykinesia, typically accompanied by resting tremor or muscle rigidity (Postuma et al. 2015).

### 2.3 Measures of the Burden of Disease

At the global, regional, and national levels, the measures of the burden of disease primarily encompass incidence, mortality, and DALYs, which are presented as the number of cases per 100,000 population and the age‐standardized rate (ASR), based on the global standardized population structure of the GBD 2021 (GBD 2021 Demographics Collaborators 2024). The estimation of each indicator (e.g., incidence, mortality, or DALYs) requires generating 1000 simulated values, with the integration of intermediate results from different etiologies and regions performed at each step of the calculation through bootstrapping (resampling with replacement). The method systematically quantifies uncertainty for all epidemiological variables, with 95% confidence intervals (CI) determined by the 2nd and 97.5th percentiles of the ranked estimates (GBD 2021 Causes of Death Collaborators 2024). DALYs are calculated by combining years of life lost (YLL) and years of life lived with disability (YLD). Specifically, YLL is calculated by multiplying the age–sex–area–year number of deaths due to PD and parkinsonism by the standardized life expectancy corresponding to each decedent's age at death. YLD is derived from the age–sex–area–year prevalence of a disease or sequelae of injury, multiplied by the corresponding disability weight (GBD 2021 Diseases and Injuries Collaborators 2024). Estimates of both components rely on life tables, disease prevalence data, and a system of disability weights. The calculation of DALYs, a core indicator of the burden‐of‐disease assessment, is described in detail in the Supporting Information of the GBD 2021 Risk Factor Study (GBD 2021 Risk Factors Collaborators 2024). The framework integrates epidemiological parameters with demographic data through a standardized process, ensuring consistency and comparability in GBD assessments.

### 2.4 SDI and Geographic Regions

The SDI serves as a composite indicator used to assess the level of development of a country or region. It is computed as the geometric mean of three dimensions: the total fertility rate of the population under 25 years of age, the average number of years of schooling for the population aged 15 and over, and lag‐adjusted per capita income. The SDI ranges from 0 to 1, with 0 representing the lowest level of schooling, the lowest per capita income, and the highest level of fertility (GBD 2021 Diseases and Injuries Collaborators 2024). In this study, 204 countries and territories were grouped into five categories (low, low‐middle, middle, high‐middle, and high) based on their SDI values to investigate the relationship between YOPD burden and socioeconomic development (Table S1). Additionally, these countries and territories were subdivided into 21 GBD regions based on geographic proximity, epidemiological characteristics, and similarities in cause‐of‐death patterns.

### 2.5 Statistical Analysis and Data Visualization

Due to differences in the age structure of populations across various countries and regions, which may influence the assessment of the YOPD burden, this study employed ASR to control for demographic differences, as well as to serve as age‐standardized measures. The burden of YOPD and its temporal variations was assessed by calculating ASR indicators for YOPD incidence, mortality, and DALYs across 204 countries and regions from 1990 to 2021.

The ASR of YOPD was calculated using the following equation:

$$\mathrm{ASR}=\frac{\sum_{i=1}^{A}a_{i}w_{i}}{\sum_{i=1}^{A}w_{i}}\times 100,000$$

where 𝑎_𝑖_ is the age‐specific rate in the i‐th age group; *w_i_* is the number of individuals in the corresponding ith age group within the standard population; and *A* is the number of age groups (Li et al. 2024).

Additionally, the trend of ASR over time was assessed by the estimated annual percentage change (EAPC). The regression equation was first established as follows: Ln(ASR) = *α* + *βX* + *ε* (where *α* represents the intercept, *X* is the year, *β* reflects the linear trend of ASR, and *ε* is the error term) (Clegg et al. 2009). The rate of change and its 95% CI were subsequently calculated using the following formula: EAPC = 100 × [exp(*β*) − 1]. Trends were classified as follows: upward trend when both the EAPC and its lower 95% CI limit were >0, downward trend when both the EAPC and its upper 95% CI limit were <0, and stable when the 95% CI range included 0 (Yin et al. 2022). The logarithmic transformation of the ASR facilitates the modeling of relative changes in rates over time and ensures that the trend follows a linear pattern. This approach is commonly employed in epidemiological studies to estimate the percent change in incidence rates over time (Hankey et al. 2000; Zhang et al. 2023). To explore the association between YOPD burden and SDI, Pearson correlation analysis and locally weighted scatterplot smoothing (LOESS) were employed to assess their nonlinear relationship with SDI (Zhang et al. 2024). Additionally, this study employed Bayesian Age‐Period Cohort (BAPC) modeling, with the integrated nested Laplace approximation (INLA) method for statistical inference. INLA was used to approximate the posterior distributions of model parameters and perform inference efficiently in the BAPC framework. Specifically, INLA was integrated into the analysis by using the INLA R package, which allowed for fast and accurate estimation of the model's marginal posterior distributions. The BAPC model, which builds upon INLA, was applied to project future trends in the global burden of YOPD from 2020 to 2050. Previous studies compared several prediction models such as the BAPC model, age‐period‐cohort model, generalized additive model, smoothed spline model, and Poisson regression, and concluded that the BAPC model had the best prediction performance (Liu et al. 2021). All data analyses and result visualizations were conducted using the open‐source software R (version 4.4.1), and the statistical significance threshold was set at *p* < 0.05. Information on the R package is provided in the Supporting Information (eMethod), along with detailed descriptions of the methods outlined above.

## 3 Results

### 3.1 Global YOPD and Its Temporal Trends

From 1990 to 2021, the global absolute numbers of new cases, deaths, and DALYs associated with YOPD consistently increased each year. Moreover, the incidence [EAPC = 1.40 (95% CI: 1.07, 1.73)] and DALY rate [EAPC = 0.14 (95% CI: 0.10, 0.18)] for the global burden of YOPD demonstrated a significant upward trend, whereas the mortality rate [EAPC = −0.42 (95% CI: −0.45, −0.39)] exhibited a downward trend, all of which were statistically significant (Table 1).

**TABLE 1:** Incidence, mortality and DALYs of young‐onset Parkinson's disease in 1990 and 2021, and change from 1990 to 2021 by age group.

Location	Number (95% UI)		Percentage change (%)	ASR, per 100,000 population (95% UI)		EAPC (95% CI)
	1990	2021		1990	2021	
**By sex groups**						
**Both sexes**						
Incidence	28,266.73 (15,154.79, 45,134.23)	81,046.67 (48,161.87, 122,328.00)	186.72	1.46 (0.79, 2.32)	2.35 (1.39, 3.55)	1.40 (1.07, 1.73)
Mortality	1335.36 (1160.20, 1470.43)	2245.68 (1995.24, 2495.67)	68.17	0.07 (0.06, 0.08)	0.06 (0.06, 0.07)	−0.42 (−0.45, −0.39)
DALYs	92,575.05 (76,441.04, 112,394.15)	180,325.32 (145,990.30, 225,031.04)	94.79	4.97 (4.13, 6.00)	5.22 (4.22, 6.52)	0.14 (0.10, 0.18)
**Men**						
Incidence	16,438.49 (8886.70, 26,034.64)	48,417.29 (28,943.52, 72,816.64)	194.54	1.67 (0.91, 2.63)	2.79 (1.67, 4.20)	1.58 (1.41, 1.75)
Mortality	832.07 (730.36, 948.29)	1475.44 (1277.38, 1685.51)	77.32	0.09 (0.08, 0.10)	0.08 (0.07, 0.10)	−0.16 (−0.20, −0.12)
DALYs	56,869.23 (46,797.10, 69,205.71)	114,178.43 (91,723.03, 141,330.67)	100.77	5.99 (4.96, 7.25)	6.57 (5.27, 8.13)	0.31 (0.26, 0.35)
**Women**						
Incidence	11,828.24 (6133.38, 19,047.20)	32,629.38 (19,138.04, 50,081.59)	175.86	1.25 (0.66, 2.00)	1.90 (1.12, 2.93)	1.21 (1.04, 1.37)
Mortality	503.29 (385.30, 585.25)	770.24 (625.27, 934.27)	53.04	0.06 (0.04, 0.07)	0.04 (0.04, 0.05)	−0.85 (−0.89, −0.81)
DALYs	35,705.82 (27,844.22, 44,898.85)	66,146.90 (51,896.51, 85,190.92)	85.26	3.92 (3.08, 4.89)	3.85 (3.01, 4.96)	−0.11 (−0.14, −0.08)
**By age group**						
**20–24 years of age**						
Incidence	617.90 (159.20, 1206.47)	812.19 (203.30, 1583.54)	31.44	0.13 (0.03, 0.25)	0.14 (0.03, 0.27)	0.35 (0.30, 0.39)
Mortality	15.27 (13.15, 17.30)	14.09 (12.76, 15.78)	−7.73	0.0031 (0.0027, 0.0035)	0.0024 (0.0021, 0.0026)	−1.03 (−1.09, −0.96)
DALYs	1209.68 (1007.36, 1449.79)	1184.41 (993.28, 1427.12)	−2.09	0.25 (0.20, 0.29)	0.20 (0.17, 0.24)	−0.80 (−0.85, −0.76)
**25–29 years of age**						
Incidence	1670.46 (432.38, 3262.24)	2383.28 (595.13, 4629.52)	42.67	0.38 (0.10, 0.74)	0.41 (0.10, 0.79)	0.31 (0.27, 0.35)
Mortality	18.0544 (15.7247, 19.6397)	21.4995 (19.2889, 24.3586)	19.08	0.0041 (0.0036, 0.0044)	0.0037 (0.0033, 0.0041)	−0.50 (−0.61, −0.39)
DALYs	2223.76 (1411.35, 3290.10)	2904.74 (1757.40, 4409.71)	30.62	0.50 (0.32, 0.74)	0.49 (0.30, 0.75)	−0.09 (−0.13, −0.05)
**30–34 years of age**						
Incidence	3023.31 (1848.53, 4429.50)	5515.86 (3415.25, 8029.88)	82.44	0.78 (0.48, 1.15)	0.91 (0.56, 1.33)	0.56 (0.54, 0.59)
Mortality	31.3465 (27.2553, 34.1156)	42.7734 (39.0203, 47.7133)	36.45	0.0081 (0.0071, 0.0089)	0.0071 (0.0065, 0.0079)	−0.67 (−0.82, −0.53)
DALYs	4535.29 (2897.22, 6719.01)	7158.21 (4495.26, 10,755.41)	57.83	1.18 (0.75,1.74)	1.18 (0.74, 1.78)	−0.01 (−0.05, 0.03)
**35–39 years of age**						
Incidence	4715.06 (1908.94, 8702.51)	9323.67 (3735.92, 16,564.28)	97.74	1.34 (0.54, 2.47)	1.66 (0.67, 2.95)	0.78 (0.75, 0.80)
Mortality	47.0260 (40.5930, 51.0218)	64.6879 (58.2505, 70.9852)	37.56	0.0134 (0.0115, 0.0145)	0.0115 (0.0104, 0.0127)	−0.54 (−0.65, −0.44)
DALYs	7999.87 (5415.93, 11,284.71)	13,700.15 (8976.07, 19,941.06)	71.25	2.27 (1.54, 3.20)	2.44 (1.60, 3.56)	0.28 (0.25, 0.30)
**40–44 years of age**						
Incidence	7515.81 (5026.32, 10,682.39)	20,478.25 (14,231.20, 28,053.98)	172.47	2.62 (1.75, 3.73)	4.09 (2.84, 5.61)	1.34 (1.14, 1.54)
Mortality	436.9754 (372.3729, 482.6116)	698.2287 (613.4897, 793.5382)	59.79	0.1525 (0.1300, 0.1685)	0.1396 (0.1226, 0.1586)	−0.40 (−0.46, −0.33)
DALYs	29,662.79 (24,954.15, 35,251.42)	52,904.56 (43,535.29, 64,618.64)	78.35	10.35 (8.71, 12.30)	10.58 (8.70, 12.92)	0.02 (−0.03, 0.06)
**45–49 years of age**						
Incidence	10,724.19 (5779.42, 16,851.11)	42,533.42 (25,981.08, 63,466.80)	296.61	4.62 (2.49, 7.26)	8.98 (5.49, 13.40)	1.88 (1.62, 2.15)
Mortality	786.6856 (691.1052, 865.7335)	1404.3983 (1252.4296, 1543.2932)	78.52	0.34 (0.30, 0.38)	0.90 (0.26, 0.33)	−0.41 (−0.47, −0.39)
DALYs	46,943.65 (40,755.03, 54,399.12)	102,473.25 (86,233.00, 123,879.11)	118.29	20.22 (17.55, 23.43)	21.64 (18.21, 26.16)	0.22 (0.15, 0.29)

*Note*: EAPC represented the annual percentage change and their 95% confidence intervals in age‐standardized rates during 30 years from 1990 to 2021.

Abbreviations: ASR, age‐standardized rate; DALYs, disability‐adjusted life‐years; EAPC, estimated annual percentage changes; SDI, sociodemographic index; UI, uncertainty interval.

The global total number of new YOPD cases increased from 28,266.73 (95% uncertainty interval [UI]: 15,154.79, 45,134.23) in 1990 to 81,046.67 (95% UI: 48,161.87, 122,328.00) in 2021, reflecting an increase of 186.72%. The number of deaths increased from 1335.36 cases (95% UI: 1160.20, 1470.43) in 1990 to 2245.68 cases (95% UI: 1995.24, 2495.67) in 2021, representing an increase of 68.17%. The number of DALYs increased from 92,575.05 cases (95% UI: 76,441.04, 112,394.15) in 1990 to 180,325.32 cases (95% UI: 145,990.30, 225,031.04) in 2021, representing an increase of 94.79% (Table 1).

In 2021, the global gender distribution of YOPD patients revealed a higher number of males compared to females, with a more significant increase in YOPD observed in males than in females. Globally, there were 48,417.29 (95% UI: 28,943.52, 72,816.64) new cases in males and 32,629.38 (95% UI: 19,138.04, 50,081.59) new cases in females (ratio_male vs. female_ = 1.48). There were 1475.44 (95% UI: 1277.38, 1685.51) male deaths and 770.24 (95% UI: 625.27, 934.27) female deaths (ratio_male vs. female_ = 1.92). The number of DALYs was 114,178.43 (95% UI: 91,723.03, 141,330.67) in males and 66,146.90 (95% UI: 51,896.51, 85,190.92) in females (ratio_male vs. female_ = 1.73). This indicates that, under the same conditions, if there are 100 new cases and 100 deaths in females, the corresponding figures for males would be 148 and 192, respectively (Table 1).

### 3.2 YOPD in SDI Regions

**FIGURE 1:**
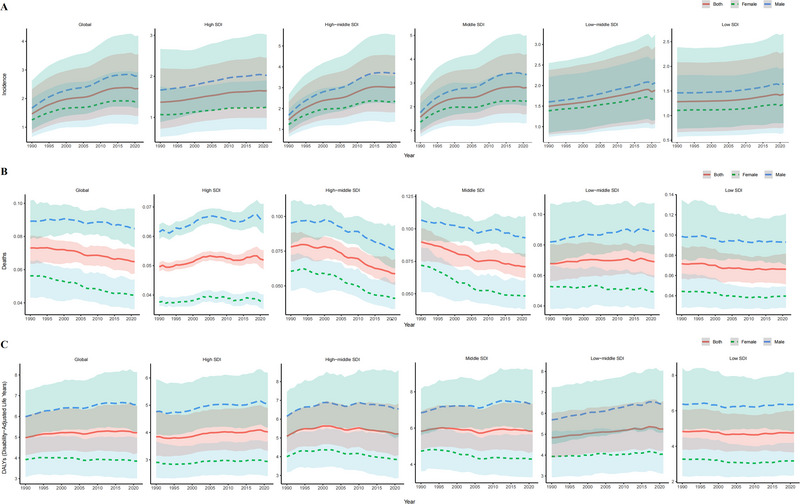
Epidemiological trends in age‐standardized rates of (A) incidence, (B) mortality, and (C) DALYs for young‐onset Parkinson's disease across 5 SDI regions, 1990–2021. SDI, sociodemographic index.

In 2021, the highest YOPD incident cases were observed in the middle SDI region (32,573.99, 95% UI: 19,657.32, 48,825.40), whereas the low SDI region had the lowest incident cases (5073.36, 95% UI: 2647.32, 8137.05). The high‐middle SDI region exhibited the highest incidence of 2.81 cases per 100,000 population. Additionally, the incidence for YOPD in all SDI regions showed an upward trend, with the most significant increase observed in the high‐middle SDI region [EAPC = 2.02 (95% CI: 1.62, 2.42)] (Table S2, Figure 1A).

Regarding the number of deaths, the middle SDI region had the highest number (829.82, 95% UI: 724.68, 947.27), whereas the low SDI region had the lowest (218.69, 95% UI: 169.81, 270.07). The middle SDI region exhibited the highest mortality at 0.07 cases per 100,000 population. Notably, from 1990 to 2021, mortality in two SDI regions showed an increasing trend (high SDI and low‐middle SDI regions), with the largest upward trend observed in high SDI region [EAPC = 0.20 (95% CI: 0.14, 0.27)], whereas mortality in three SDI regions showed a decreasing trend, with the most significant decrease observed in the high‐middle SDI region [EAPC = −1.07 (95% CI: −1.18, −0.96)]. All of these trends were statistically significant (Table S2, Figure 1B).

Regarding DALYs, the middle SDI region had the highest number (67,961.50, 95% UI: 54,799.33, 85,364.52), whereas the low SDI region had the lowest (16,030.26, 95% UI: 12,206.05, 20,541.65). The middle SDI region exhibited the highest DALY rate at 5.83 cases per 100,000 people. From 1990 to 2021, the DALY rate in two SDI regions showed an increasing trend (high SDI and low‐middle SDI regions), with the largest upward trend observed in the low‐middle SDI region [EAPC = 0.32 (95% CI: 0.29, 0.34)], whereas DALY rate in three SDI regions showed a decreasing trend, with the most significant decrease observed in the low SDI region [EAPC = −0.08 (95% CI: −0.13, −0.03)]. All of these trends were statistically significant (Table S2, Figure 1C).

In summary, the middle SDI region presents the most significant public health challenges, with the highest absolute values for all three indicators—new cases, deaths, and DALYs—among all SDI regions. Meanwhile, the middle SDI and high‐middle SDI regions showed the most substantial rise in incidence, mortality, and DALY rate, which are the key components contributing to the growing global YOPD burden. Notably, the low SDI region exhibited considerably lower values than the other regions in all burden‐of‐disease indicators.

### 3.3 YOPD in Different GBD Regions

**FIGURE 2:**
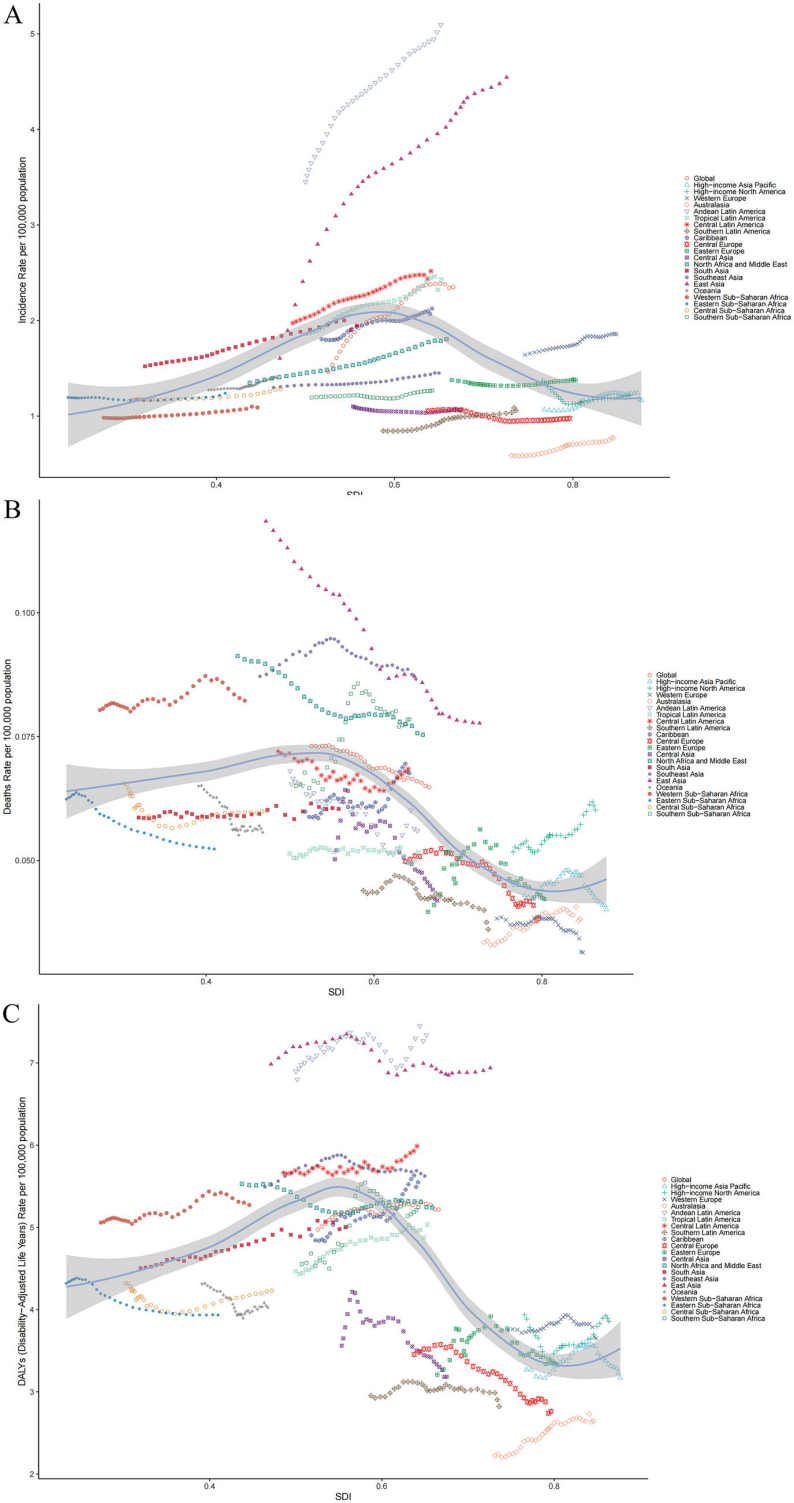
Age‐standardized rates of (A) incidence, (B) mortality, and (C) DALYs of young‐onset Parkinson's disease across 21 GBD regions by SDI, 1990–2021. DALYs, disability‐adjusted life‐years.

In 2021, among the 21 GBD regions, East Asia (34,920.25 cases) reported the highest number of new YOPD cases, followed by South Asia (15,224.84 cases) and North Africa and the Middle East (5,121.91 cases), collectively accounting for nearly 70% of the global new YOPD cases. The remaining regions each had fewer than 5,000 cases, with Oceania reporting the lowest (73.07 cases). Additionally, Andean Latin America exhibited the highest incidence at 5.1 cases per 100,000 population, followed by East Asia and Central Latin America, with 4.54 and 2.52 cases per 100,000 population, respectively, whereas Australasia reported the lowest incidence at 0.77 cases per 100,000 population. From 1990 to 2021, incidence trends varied across regions, with 17 GBD regions showing an upward trend, and East Asia exhibiting the greatest increase in incidence [EAPC = 2.47 (95% CI: 2.05, 2.89)]. Meanwhile, four regions exhibited a decreasing trend, with Central Europe showing the most significant decrease [EAPC = −0.40 (95% CI: −0.74, −0.07)], all of which were statistically significant (Table S2, Figure 2A).

The highest number of YOPD deaths was once again observed in East Asia (593.18 cases), followed by South Asia (460.29 cases) and Southeast Asia (285.47 cases). These three GBD regions accounted for 60% of the total YOPD deaths globally, whereas Oceania had the lowest number, with only 2.85 cases. Additionally, Southeast Asia exhibited the highest mortality at 0.09 cases per 100,000 population, whereas Western Europe reported the lowest at 0.03 cases per 100,000 population. From 1990 to 2021, six regions exhibited an increasing trend, with Southern Sub‐Saharan Africa experiencing the highest rise in mortality [EAPC = 0.73 (95% CI: 0.47, 0.99)], whereas the majority of regions, a total of 15, showed a decreasing trend, with East Asia showing the most significant decrease [EAPC = −1.47 (95% CI: −1.55, −1.39)]. All of these trends were statistically significant (Table S2, Figure 2B).

Additionally, East Asia recorded the highest number of YOPDDALYs (53,088.71 cases), followed by South Asia (38,478.66 cases) and Southeast Asia (18,458.46 cases). These three GBD regions accounted for 60% of the global total YOPDDALYs, whereas Oceania reported the lowest number, at 207.77 cases. Andean Latin America exhibited the highest DALY rate at 7.34 cases per 100,000 population, whereas Australasia reported the lowest at 2.65 cases per 100,000 population. A total of 12 GBD regions exhibited an increasing trend from 1990 to 2021, with Australasia showing a significant rise in DALY rate [EAPC = 0.71 (95% CI: 0.62, 0.79)], and nine regions showed a decreasing trend, with Central Europe experiencing the most significant decrease [EAPC = −0.88 (95% CI: −0.97, −0.79)]. The most pronounced decrease was observed in Central Europe [EAPC = −0.98 (95% CI: −0.97, −0.80)], all of which were statistically significant (Table S2, Figure 2C).

Notably, the GBD regions in Asia represent a significant portion of the YOPD burden of disease, with East Asia, South Asia, and Southeast Asia collectively constituting the primary burden regions. Additionally, Figure 2 illustrates the incidence, mortality, and DALY rate of YOPD across GBD regions from 1990 to 2021, along with their relationships to SDI. Incidence, mortality, and DALY rate exhibited an approximately inverted U‐shaped relationship with SDI, peaking at an SDI value of 0.55–0.60, and subsequently declining as SDI levels increased, reaching a minimum at SDI values between 0.80 and 0.85. Furthermore, mortality and DALY rate, after reaching their lowest points, gradually increased again as SDI levels rose. The fitted line suggests that the greatest YOPD burden is observed in the low‐middle SDI region.

### 3.4 YOPD in Different Countries and Regions

**FIGURE 3:**
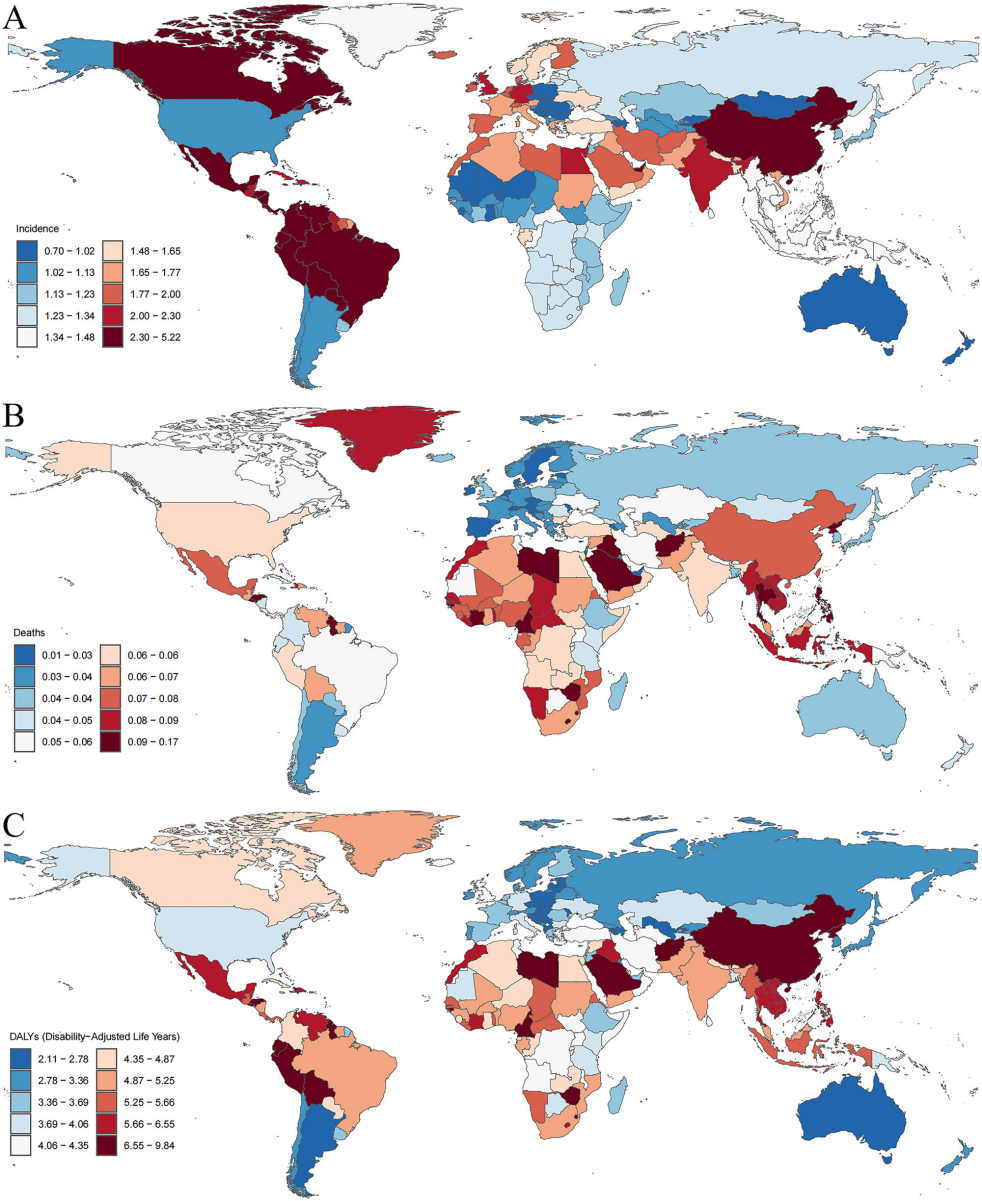
Age‐standardized rates of (A) incidence, (B) mortality, and (C) DALYs of young‐onset Parkinson's disease in 204 Countries and Territories, 1990–2021.

In 2021, China had the highest number of new YOPD cases among 204 countries, accounting for nearly half of the global total (34,314.28/81,046.67 = 42.34%). India (12,395.28 cases) and Brazil (2576.90 cases) followed, collectively accounting for 60.81% of the global total YOPD cases. Peru had the highest YOPD incidence at 5.17 cases per 100,000 population, followed by Bolivia (5.13) and Ecuador (4.92) cases per 100,000 population, respectively. These three countries collectively accounted for 60.81% of the global total of YOPD cases. Between 1990 and 2021, 169 countries or territories showed a significant increase in incidence, with Norway showing the largest increase (EAPC = 2.63, 95% CI: 2.47, 2.79), followed by China and the Democratic People's Republic of Korea. Only 23 countries or territories showed a decreasing trend in incidence, with Poland experiencing the largest decrease (EAPC = −1.23, 95% CI: −1.53, −0.93), followed by Ethiopia and the United States of America (Figure 3A).

In 2021, the countries reporting the highest number of YOPD‐related deaths were China and India, while Saudi Arabia exhibited the highest mortality burden, with a rate of 0.17 cases per 100,000 population. This was followed by Afghanistan and Guinea‐Bissau, with rates of 0.16 and 0.14 per 100,000 population, respectively. Between 1990 and 2021, 61 countries or territories exhibited a significant upward trend in mortality, with Lesotho displaying the largest increase [EAPC = 2.74 (95% CI: 2.20, 3.28)]. A total of 121 countries or territories displayed a downward trend in mortality, with the United Arab Emirates showing the largest decrease [EAPC = −3.22 (95% CI: −3.77, −2.66)] (Figure 3B).

In 2021, in terms of the DALY burden, China (51,112.70 cases) and India (30,974.66 cases) remained the top two countries globally, whereas all other countries or territories reported fewer than 10,000 DALY cases. Saudi Arabia exhibited the highest DALY rate, with 9.75 cases per 100,000 population, followed by Afghanistan and the Democratic People's Republic of Korea, with rates of 9.25 and 8.98 cases per 100,000 population, respectively. Between 1990 and 2021, 74 countries or territories exhibited a significant upward trend in DALY rate, with the largest increase observed in Lesotho [EAPC = 2.00 (95% CI: 1.64, 2.36)]. In contrast, 97 countries or regions displayed a decreasing trend in DALY rate, with the largest decrease observed in Rwanda [EAPC = −1.94 (95% CI: −2.24, −1.65)] (Figure 3C).

Overall, the distribution of the YOPD burden across 204 countries or regions exhibited considerable heterogeneity, but the geographic patterns were consistent with those observed in the GBD regional analyses. In 2021, China and India were the leading countries globally in terms of YOPD incidence, mortality, and DALY burden. Additionally, the high‐middle SDI region exhibited a higher YOPD burden, with countries such as Saudi Arabia, which had the highest mortality and DALY rate globally, displaying a prominent burden of disease. Furthermore, Norway exhibited the largest increase in incidence, whereas Lesotho observed the most significant increase in mortality and DALY rate globally. Notably, although China's incidence showed an increasing trend (EAPC = 2.47), its mortality decreased substantially (EAPC = −1.47), highlighting the paradoxical trend of rising cases and declining deaths.

### 3.5 Age and Sex Differences in the Burden of YOPD

**FIGURE 4:**
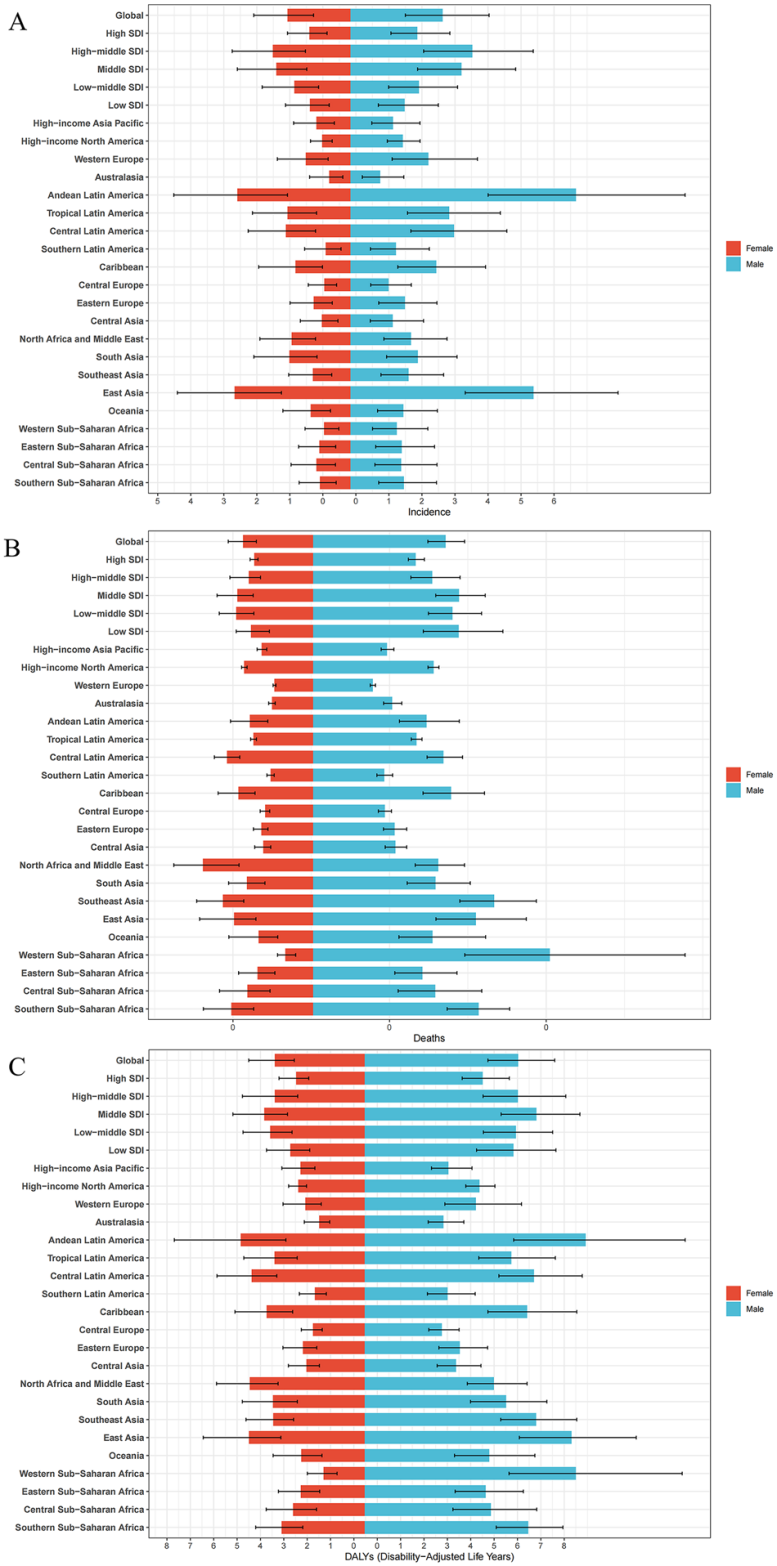
Age‐standardized rates of (A) incidence, (B) mortality, and (C) DALYs of young‐onset Parkinson's disease in 2021, by sex and region.

From 1990 to 2021, global YOPD exhibited a distinct age‐related pattern, with incidence, mortality, and DALY rates all positively correlated with advancing age. Among all age groups, only the 20–24 age group exhibited a reduction in the absolute number of deaths and DALYs, whereas the 45–49 age group experienced the most significant increase in the overall YOPD burden. Notably, all age groups exhibited a statistically significant downward trend in mortality, with the most significant decrease observed in the 20–24 age group [EAPC = −1.03 (95% CI: −1.09, −0.96)]. Additionally, the 35–39 age group experienced the most significant increase in DALY rate [EAPC = 0.28 (95% CI: 0.25, 0.30)], whereas the 45–49 age group exhibited the largest increase in incidence [EAPC = 0.22 (95% CI: 0.15, 0.29)] (Table 1). In the gender analysis, males exhibited higher global incidence, mortality, and DALY rates than females, with the largest gap observed in the 45–49 age group (Figure S1). In 2021, the high‐middle SDI region exhibited the highest YOPD incidence for both sexes, and Andean Latin America recorded the highest incidence for both sexes (Figure 4A). Mortality rates were highest in Southeast Asia for both sexes, although variation across SDI strata was noted (Figure 4B). Similarly, DALY rates were highest in Andean Latin America, and middle SDI regions also bore a substantial burden (Figure 4C). Overall, men experienced approximately 1.5–2.1 times the incidence, mortality, and DALYs burdens compared with women.

### 3.6 Future Forecasts of Global Burden of YOPD

**FIGURE 5:**
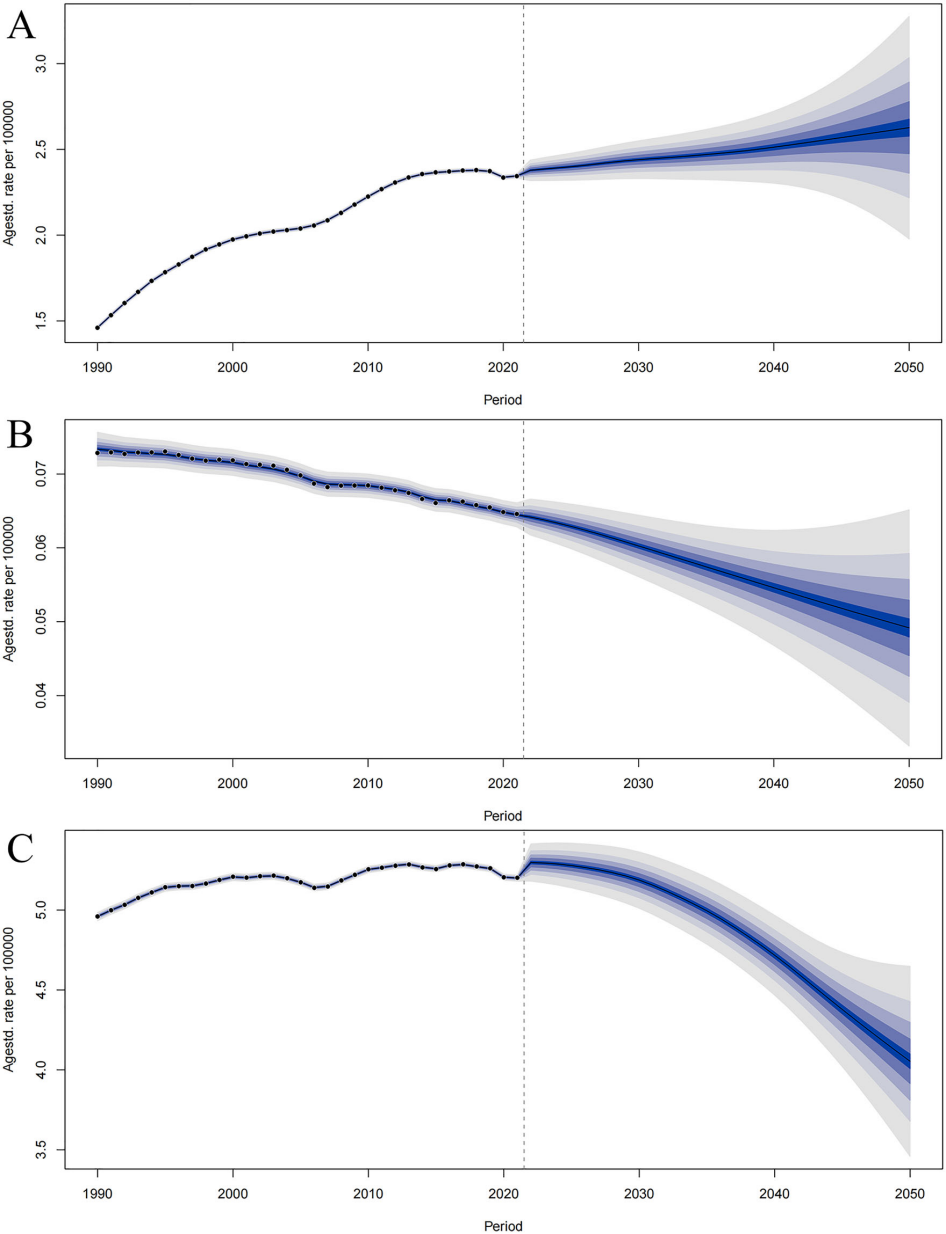
The projected temporal trends in the global burden of age‐standardized rates of (A) incidence, (B) mortality, and (C) DALYs for young‐onset Parkinson's disease, 2021–2050. The black line shows the global level. Shading indicates the upper and lower limits of the 95% UI.

Using the YOPD data from GBD 2021, we applied the BAPC model to predict the temporal trends of incidence, mortality, and DALY rate for global YOPD from 2021 to 2050. The results indicated that the incidence of YOPD is expected to increase over the next 30 years, whereas the mortality and DALY rate are expected to decrease significantly. The overall incidence of YOPD is projected to gradually rise from 2021 to 2050, reaching 2.63 cases per 100,000 people by 2050, representing a 12.39% increase compared to 2021 (Figure 5A). In contrast, mortality and DALY rate for YOPD are expected to decrease significantly, reaching 0.049 cases per 100,000 people and 4.05 cases per 100,000 people, respectively, by 2050, representing a decrease of 23.75% and 22.05% from the 2021 levels (Figure 5B,C). Additionally, the rising incidence and declining mortality and DALY rate trends across all age groups closely mirrored the overall trend, suggesting that the evolutionary pattern is generalizable across age groups (Figure S2). The projected curves revealed a biphasic evolution of global YOPD, with the number of YOPD cases projected to reach 100,000 by 2050, whereas the number of attributable DALYs is expected to decrease by 17,000 during the same period.

### 3.7 Factors Associated With EAPC

**FIGURE 6:**
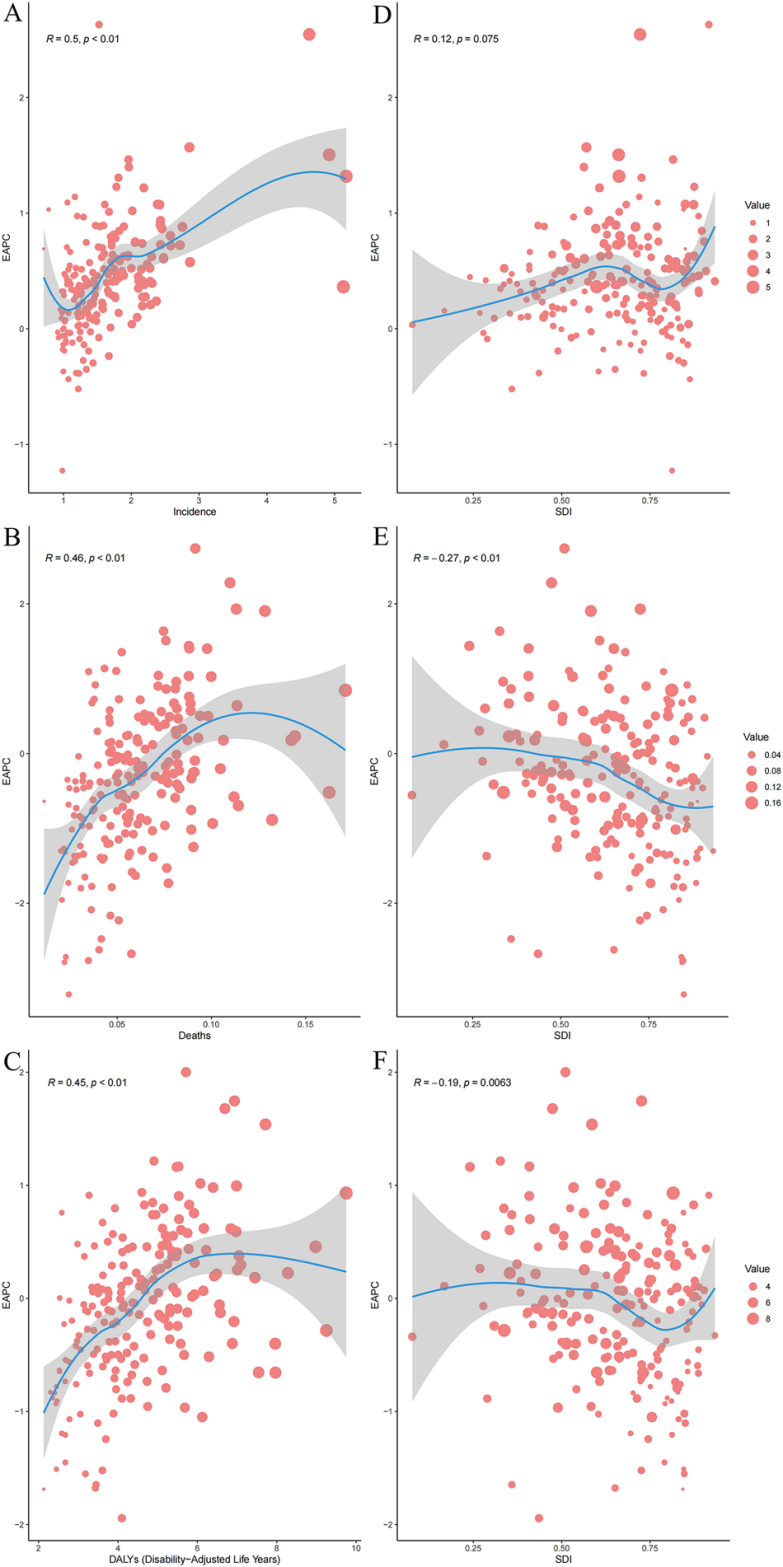
The correlation between EAPC and young‐onset Parkinson's disease indicators (incidence rate, mortality rate, DALYs) and SDI in 2021. The analysis includes: (A) correlation between EAPC and incidence rate, (B) correlation between EAPC and mortality rate, (C) correlation between EAPC and DALYs of young‐onset Parkinson's disease, and (D–F) correlations between EAPC and SDI in 2021. Each dot represents one country, and the solid blue line denotes the fitted regression trend with a 95% confidence interval. The circles represent countries with available SDI data. The size of the circle increases with the number of YOPD cases. Outliers represent countries with extreme values in either incidence or SDI, which may influence the regression analysis. These outliers should be considered with caution in further interpretation of the results. EAPC, estimated annual percentage change.

In 2021, EAPC exhibited a moderate positive association with YOPD incidence (*R* = 0.5, *p* < 0.01), whereas no significant association was found between EAPC and SDI in YOPD‐related incidence (*R* = 0.12, *p* = 0.075). Furthermore, EAPC exhibited a moderate positive correlation with YOPD mortality (*R* = 0.46, *p* < 0.01), while showing a weak negative correlation with SDI (*R* = −0.27, *p* < 0.01). A similar correlation pattern was observed in the DALYs indicator, where EAPC was moderately positively correlated with the YOPD DALY rate (*R* = 0.45, *p* < 0.01), whereas a weak negative correlation was found between EAPC and SDI in the DALYs indicator (*R* = −0.19, *p* = 0.0063) (Figure 6).

## 4 Discussion

This study offers a comprehensive analysis of temporal trends, regional differences, age‐sex disparities, and future projections of the global YOPD burden of disease, aspects that have often been overlooked in previous PD‐related research. Over the past three decades, the global number of YOPD incident cases has more than tripled, whereas the number of deaths and DALYs has doubled. There has been a general increase in the global YOPD burden of disease (Ben‐Shlomo et al. 2024). From 1990 to 2021, the incidence and DALY rate for YOPD increased significantly worldwide, whereas the mortality declined. The middle SDI and high‐middle SDI regions ranked among the highest in terms of increases in incidence, mortality, and DALY rate, whereas the low‐middle SDI region bears the greatest YOPD burden. The burden of YOPD was consistently higher in men than in women, with incidence, mortality, and DALYs ratios of 1.48, 1.92, and 1.73, respectively, peaking in the 45–49 age group. Our findings highlight the paradoxical phenomenon of increasing incidence and decreasing mortality, alongside significant regional and sociodemographic heterogeneity and persistent gender differences. The productivity loss caused by YOPD patients far exceeds that of LOPD, owing to the early age of onset, prolonged disease duration, and complex social roles. Furthermore, the substantial regional and gender disparities in the global burden underscore its wide‐ranging impact and potential for control, suggesting an urgent need for targeted healthcare efforts to address the growing YOPD burden.

In this study, we observed variations in burden across countries, with low‐middle SDI nations bearing the greatest burden. In particular, the three GBD regions in Asia—East Asia, South Asia, and Southeast Asia—are central to the YOPD burden of disease, comprising 60% of the global YOPD burden. This contrasts with past findings that the prevalence of PD in East Asia is typically lower than in Europe and North America (Lim et al. 2019). Recent studies have shown that PD has been rapidly increasing in several Asian countries, particularly in China, Korea, Japan, Thailand, and Israel, aligning with the regional burden distribution findings of the current study (Mahmood et al. 2021). This aligns with the theory of “epidemiological transition,” wherein middle‐income countries are experiencing a rising burden of NCDs due to urbanization, environmental degradation, and inadequate healthcare infrastructure (GBD 2021 Diseases and Injuries Collaborators 2024), resulting from limited resources. Asia, home to nearly 60% of the global population, with countries like China and India, contributes directly to the high absolute number of cases. Even with similar incidence rates to other regions, the large population base exacerbates the total burden. East Asian countries, such as Japan and South Korea, are experiencing the fastest aging rates globally, with the risk of PD increasing significantly with age (Luo et al. 2024; Chhetri et al. 2023).

Although YOPD refers specifically to early onset cases, the aging of societies increases overall pressure to prevent and control neurodegenerative diseases. This demographic shift may indirectly affect how resources are allocated for the early onset group. In Asia, rapid urbanization has worsened YOPD prevalence. Environmental pollutants play a key role in this trend. These pollutants include industrial chemicals, air pollution–related particulate matter, heavy metals, various occupational exposures, and pesticide use (Aravindan and Newell 2024). Furthermore, the LRRK2 G2385R and R1628P mutations occur far more frequently in Asian populations than in Caucasian populations. These mutations are associated with a higher risk of PD and an earlier age of onset (Xiao et al. 2018, 2022; Bandres‐Ciga et al. 2020). Additionally, certain GBA mutations and other rare genetic drivers found in Asians may promote abnormal aggregation of α‐synuclein. This aggregation accelerates the neurodegenerative process in PD (Kim et al. 2020; Andrews et al. 2024). According to the third edition of the World Health Organization (WHO) Global Report on Prevalence Trends in Tobacco Use (2000–2025), tobacco use is most prevalent in the WHO Southeast Asia Region (World Health Organization 2019). Some studies suggest that smokers have approximately a 40% lower risk of developing PD (Rossi et al. 2018). As global smoking bans have been implemented over the years, smoking prevalence in Asia is decreasing dramatically, which may lead to a further increase in PD cases (Rossi et al. 2018; GBD 2019 Tobacco Collaborators 2021). Additionally, this is associated with advancements in medical testing technologies and more widespread disease screening methods, such as neuroimaging and AI modeling, potentially leading to more YOPD diagnoses (Ye et al. 2025; Pagan 2012; Dentamaro et al. 2024). Notably, between 1990 and 2021, the burden of disease in the high SDI region grew significantly more than the global average, likely due to the greater human and financial resources in developed countries, which contribute to higher rates of early detection and improved treatment outcomes for YOPD (Valasaki 2023; Daida et al. 2024). This phenomenon may also be linked to more advanced medical practices and improved access to care in these regions. This suggests that deficiencies remain in disease control and management efforts in these regions, indicating that future health initiatives should consider the existing and potentially growing burden of regionally differentiated YOPD.

Globally, the burden of YOPD differs significantly by gender and age. This is in line with the global epidemiology of PD, where male dominance is well established (Luo et al. 2024). Possible explanations include the neuroprotective effects of estrogen and other gender‐related genetic factors in women (Chen et al. 2023), differential exposure to occupational hazards (Tanner et al. 2009; Frigerio et al. 2005), delayed diagnosis in female PD patients due to differences in symptom expression and healthcare‐seeking behaviors (Savica et al. 2017), and the higher pressures faced by males to advance in education and meet societal expectations (Sieurin et al. 2018). Notably, the gender gap widens with age, reaching its peak at 45–49 years, which may reflect cumulative environmental exposures or hormonal changes during midlife (Schaffner et al. 2025). These findings highlight the need for gender‐sensitive interventions, particularly in high‐burden regions such as the high‐middle SDI region and Andean Latin America, where the male‐to‐female incidence ratio exceeds 2:1.

In line with the traditional age‐dependent onset trend for PD, the burden of YOPD rises with age, peaking at 45–49 years, in accordance with the typical onset window (21–50 years) (Pringsheim et al. 2014). The significant rise in incidence in this age group suggests that an increasing proportion of the population is at risk of long‐term disability, with implications for global workforce productivity and caregiver burden (Leite Silva et al. 2023; McDaniels et al. 2023). Some studies have shown that indirect costs (e.g., loss of productivity) are two to three times higher for individuals with YOPD compared to those with LOPD, primarily due to a diminished ability to work and an increased need for family caregiving (Martinez‐Martin et al. 2019). Strikingly, mortality declined across all age groups, with the most significant decrease observed in the 20–24 age group (EAPC = −1.03). This may be attributed to the fact that the younger generation generally enjoys better living conditions and a higher level of health awareness compared to older generations. These disparities can be partly attributed to superior education levels among the younger generation, as well as improved living conditions (Chen et al. 2022; Osler et al. 2022). However, the increase in DALY rate among individuals aged 35–39 years (EAPC = 0.28) suggests that surviving patients face a cumulative burden of disability, underscoring the need for rehabilitation and social support services. It is important to note that most patients with PD do not die directly from the disease. Instead, complications such as lung infections caused by PD or unintentional injuries, such as falls, may elevate the risk of death in PD patients to some extent (Bloem et al. 2021; Karceski 2018). Frequently, patients with YOPD encounter unique social, professional, and personal challenges that are often overlooked by general PD organizations (Domingos et al. 2023). Thus, it is equally important to emphasize the prevention and treatment of PD‐related complications.

Additionally, this study forecasts that by 2050, global YOPD cases will surpass 100,000, driven by ongoing increases in incidence. Despite declines in both mortality (−23.75%) and DALY rate (−22.05%), the number of new YOPD cases will surpass the absolute reduction in DALYs, leading to a net increase in health losses. This “dual trajectory” underscores the significance of YODP projections, wherein treatment advances reduce mortality but extend dependence. Policymakers must prepare for the growing demand for long‐term care, disability benefits, and mental health services, particularly in the high‐middle SDI region with the fastest growing incidence and Asian hotspots with the highest YOPD burdens, where priority screening programs are most urgently needed. In addition, although a positive correlation was observed between baseline incidence and the EAPC in incidence, this finding remains exploratory and may arise from a “diagnostic buffer” effect caused by insufficient early detection, as well as cumulative risk factors in regions with high prevalence. Regarding the SDI, there was no significant impact on morbidity trends, likely because regions with high SDI possess both superior screening systems and more effective preventive measures that counterbalance each other; conversely, with respect to mortality and DALYs, high SDI countries benefit from more abundant medical resources and rehabilitation services, resulting in a modest inverse correlation. Future research should incorporate more nuanced macro‐level indicators (e.g., healthcare expenditure ratios and health insurance coverage) and microlevel factors (e.g., individual risk exposures and genetic susceptibility) to more thoroughly investigate the determinants of changes in the global burden of YOPD across countries.

Although this study presents a global overview of the YOPD burden using the most recent GBD 2021 data, several limitations exist. First, the GBD database includes global data, and discrepancies in the definition and reporting of YOPD may exist across countries, particularly in studies based on death certificates, where underreporting is a recognized issue (von Campenhausen et al. 2005). Underdiagnosis in the low SDI region may lead to an underestimation of the true burden, particularly among women and rural populations. Consequently, the number of YOPD cases in each region may be either overestimated or underestimated, representing a significant source of potential bias. Second, the absence of relevant studies or data limits our ability to fully explain the reasons behind trends in some countries. Another limitation arises from the GBD database's use of age groups based on 5‐year intervals, restricting the inclusion criteria for YOPD age categories. Finally, it is important to acknowledge that the risk factors associated with YOPD in GBD 2021 are limited and do not capture the full spectrum of disease etiologies. As such, we have not systematically analyzed risk factors, which creates limitations in developing targeted interventions and prevention strategies. This underscores the need for future iterations of the GBD program to incorporate a broader range of relevant risk factors. Additionally, the analyses in this study were based on pooled data, with the GBD database serving as the sole data source, unlike other global databases such as WHO's Global Health Estimates, which compiles data from multiple sources. Cohort studies based on large samples may provide more detailed results. Nevertheless, this study represents the first comprehensive assessment of global YOPD burden trends over the last 30 years, laying the foundation for future research to address existing gaps and improve the quality and comparability of global YOPD data.

## 5 Conclusion

This study provides a systematic analysis of the global YOPD burden‐of‐disease trends and future projections from 1990 to 2021. The global burden of YOPD continues to rise, driven by factors such as population aging, increased environmental risks, and unequal healthcare resource distribution. The study highlights a key epidemiologic paradox: Although overall mortality declines, incidence and disability continue to rise. This paradox suggests that, despite advances in medical technology reducing mortality and incidence, significant gaps remain in disease prevention efforts. The current situation calls for urgent global action, including the establishment of regional prevention and control programs, the implementation of targeted measures, the development of gender‐sensitive interventions, and strengthening neurodegenerative disease surveillance networks in low‐ and middle‐income countries. By implementing these strategic initiatives, the international community can effectively curb the spread of YOPD and significantly improve the long‐term quality of life for patients and their families worldwide. Achieving this goal requires balancing healthcare resource allocation while strengthening environmental risk management and responses to aging.

## Author Contributions

**Yanjie Jiang**: conceptualization, methodology, writing – review and editing, writing – original draft, formal analysis, supervision. **Hanyu Wang**: methodology, conceptualization, writing – original draft, writing – review and editing. **Xingyi He**: conceptualization, writing – original draft, writing – review and editing, software. **Rui Fu**: investigation, validation, formal analysis. **Zhihui Jin**: conceptualization, writing – original draft. **Qinwei Fu**: conceptualization, writing – review and editing. **Xinlang Yu**: conceptualization, validation, methodology. **Wenshan Li**: conceptualization, writing – review and editing. **Xiaoyu Zhu**: software, data curation. **Shipeng Zhang**: conceptualization, methodology, writing – review and editing, writing – original draft. **Yan Lu**: conceptualization, methodology, writing – review and editing, writing – original draft.

## Ethics Statement

The authors have nothing to report.

## Consent

The authors have nothing to report.

## Conflicts of Interest

The authors declare no conflicts of interest.

## Peer Review

The peer review history for this article is available at https://publons.com/publon/10.1002/brb3.70659.

## Supporting information




**Supplementary Material**: brb370659‐sup‐0001‐SuppMat.docx

## Data Availability

The data sets generated and/or analyzed during the current study are available in the GBD repository (https://vizhub.healthdata.org/gbd‐results/).
